# Glypican-3 Targeting Immunotoxins for the Treatment of Liver Cancer

**DOI:** 10.3390/toxins8100274

**Published:** 2016-09-22

**Authors:** Bryan D. Fleming, Mitchell Ho

**Affiliations:** Laboratory of Molecular Biology, Center for Cancer Research, National Cancer Institute, National Institutes of Health, Bethesda, MD 20892, USA; Bryan.Fleming@nih.gov

**Keywords:** recombinant immunotoxin, glypican-3 (GPC3), hepatocellular carcinoma, liver cancer, monoclonal antibodies, pseudomonas exotoxin

## Abstract

Hepatocellular carcinoma (HCC) is the most common form of primary liver cancer, yet no effective therapeutics exist. This review provides an overview of the recent development of recombinant immunotoxins for the treatment of glypican-3 (GPC3) expressing HCC. GPC3 is a cell surface heparan sulfate proteoglycan that is overexpressed in HCC, but is absent from normal adult human tissues. Treatment of HCC with anti-GPC3 immunotoxins represents a new therapeutic option. Using phage display and hybridoma technologies, three high affinity antibodies (HN3, HS20 and YP7) have been generated against GPC3. Two of these antibodies (HN3 and HS20) have demonstrated the ability to inhibit Wnt/Yap signaling, leading to a reduction in liver cancer cell proliferation. By combining the HN3 antibody capable of inhibiting Wnt/Yap signaling with the protein synthesis inhibitory domain of the Pseudomonas exotoxin, a recombinant immunotoxin that exhibits a dual inhibitory mechanism was generated. This immunotoxin was found to be highly effective in the treatment of human HCCs in mouse xenograft models. Engineering of the toxin fragment to reduce the level of immunogenicity is currently being explored. The development of immunotoxins provides opportunities for novel liver cancer therapies.

## 1. Introduction 

The emergence of antibody-based therapeutics has been met with great success when used to treat cancer. Monoclonal antibodies can work in numerous ways to promote anti-cancer effects. Antibodies can help to activate the immune response by promoting Antibody Dependent Cell Cytotoxicity (ADCC) and Complement Dependent Cytotoxicity (CDC) [1,2]. Recent reports have indicated that antibodies can function as checkpoint inhibitors to promote the activation of cytotoxic T cells [3,4]. Antibody therapies can also inhibit cancer cell proliferation by blocking the binding of growth factors [2]. Muromonab and rituximab were the earliest monoclonal antibodies to demonstrate anti-cancer effects. These antibodies have been used to treat blood cancers including T cell acute lymphoblastic leukemia and non-Hodgkin’s lymphoma [5,6,7,8,9,10]. The ability of antibodies to inhibit cancer growth by different mechanisms allows them to be applicable for various cancers.

There were over fifty monoclonal antibody therapeutics being evaluated in Phase III clinical trials in 2015 [11]. Twelve of the antibodies in Phase III trials were being evaluated as cancer therapeutics [11]. There were two anti-cancer antibodies approved by the FDA in 2015; dinutuximab that targets the GD2 disialoganglioside is used to treat neuroblastoma [11,12] and daratumumab targeting CD38 is used for multiple myeloma [11,13]. While monoclonal antibody treatments are proving beneficial in cancer therapy, they are not without their downsides. Administration of rituximab and muromonab can trigger a cytokine release syndrome and can even result in opportunistic viral infections [14]. Additionally, antibody therapeutics have been associated with hypersensitivity reactions that can cause headache, diarrhea, fever, and hypotension [15,16]. If untreated, these reactions can develop into anaphylaxis and serum sickness, both of which can be life threatening [16]. Despite the potential side effects, monoclonal antibody therapeutics have changed the landscape of cancer therapy.

The field of antibody therapeutics has expanded beyond simple monoclonal antibodies. New forms of antibody-based therapeutics include antibody drug conjugates (ADC), chimeric antigen receptor T cells (CAR-T), and recombinant immunotoxins (RIT). Antibody drug conjugates use the specificity of the antibody to target chemotherapeutic drugs directly to cancerous cells. Brentuximab vedotin (Anti-CD30, MMAE) and ado-trastuzumab emtansine (Anti-Her2/neu, maytansine) are approved by the FDA for the treatment of Hodgkin’s lymphoma and HER2 positive metastatic breast cancer, respectively [17,18,19,20]. These drugs work as mitosis inhibitors by blocking tubulin polymerization [17,20]. These treatments have an advantage over standard chemotherapies because the targeting of drugs helps to reduce off-target side effects [17,21]. It is important to note that a similar class of therapeutics are the antibody radioisotope conjugates. These function off the same principle as the ADC, but use radioisotopes to damage DNA rather than chemicals [21]. Chimeric antigen receptor T cells represent the newest class of cancer therapeutics. T cell activation is highly regulated, requiring the activation of the T cell receptor by major histocompatibility complex (MHC) displaying peptide and the activation of CD28 by costimulatory molecules on antigen presenting cells [22]. By fusing an antibody binding domain with important T cell signaling domains (CD28 and CD3ζ), the requirement for T cells to interact with MHC is removed. Several CAR-T based therapies are currently in clinical trials, but none have received FDA approval. While all of these therapeutic classes have potential, this review will focus on the generation of recombinant immunotoxins.

Recombinant immunotoxins are fusion proteins that combine the antigen binding domain of an antibody with a bacterial toxin like Pseudomonas exotoxin A. These bacterial toxins have the ability to inhibit cellular protein synthesis through the modification of elongation factors. Advances in recombinant immunotoxins over the last thirty years is largely due to a better understanding of the toxin portion. Structural studies on Pseudomonas exotoxin A has revealed the existence of three domains. Domain III has been studied the most and was found to be responsible for the catalytic activity of the toxin [23,24,25]. This domain inhibits protein synthesis by modifying elongation factor 2 through ADP-Ribosylation [24,26,27]. The blocking of protein synthesis can lead to the initiation of apoptotic death in cancer cells [24,26]. The C-terminal amino acid sequence in domain III is thought to play a role in retrograde targeting of the toxin from the Golgi apparatus to the endoplasmic reticulum. It contains a REDL sequence that is capable of binding to the mammalian KDEL receptor [24,28,29,30]. Domain I is believed to initiate cell surface attachment by binding to CD91 (α_2_-macroglobulin receptor) [25,31]. In most recombinant immunotoxin designs, domain I is removed and replaced by the antibody fragment. This substitution allows for the targeting of immunotoxins to a diverse array of cancer antigens. Understanding the function of domain II has been more difficult than the other domains. It was originally believed to be involved in the translocation of the toxin through the membrane into the cytosol [26]. However, research on this domain has provided evidence that this may not be the case. It is now believed that the toxin fragment may gain entry to the cytosol by moving through the Sec61p membrane channel and the endoplasmic reticulum-associated protein degradation (ERAD) pathway [24,32,33,34]. The low number of lysine residues in the cytosolic fraction may help the protein to avoid ubiquitination, which in turn helps it to escape degradation in the ERAD pathway [34]. Research on immunotoxins has helped to identify the presence of a furin cleavage site in domain II. Cleavage at this site has been shown to be partially responsible for separating the toxin fragment from the antibody portion [35,36]. A better understanding of how the toxin functions has allowed for the development of more potent therapeutics.

Recombinant immunotoxins are being used in the clinical setting to treat several forms of cancer. Clinical trials involving anti-CD22 and anti-mesothelin immunotoxins are ongoing. The anti-CD22 immunotoxin was constructed by fusing an antibody Fv region with a truncated form of Pseudomonas exotoxin A. This therapeutic was used with high success in the treatment of hairy cell leukemia [27,37]. A clinical trial published in 2012 demonstrated that patients with relapsed or refractory hairy cell leukemia responded positively to immunotoxin treatment. Of the twenty-eight patients included in the study, thirteen showed complete remission and eleven showed partial remission [38]. The anti-mesothelin immunotoxin, SS1P, has been tested on patients diagnosed with a variety of mesothelin expressing solid tumors [39]. Mesothelioma, ovarian, and pancreatic cancers were included in a study that determined SS1P was well tolerated by patients and that treatment reduced the rate of disease progression [40,41].

Other immunotoxins are currently in preclinical development to target cancer. Immunotoxins have been used to treat intracranial tumors in rodent models by targeting the EGF receptor expressed in glioblastomas [42]. Similarly, anti-c-Met and anti-epithelial cell adhesion molecule (EpCAM) have been evaluated for their ability to target gastric cancer and a wide range of solid tumors including breast, colon, and liver cancers, respectively [43,44,45]. Instead of substituting the antibody portion to make new immunotoxins, it is also possible to substitute the toxin fragment. A diphtheria toxin based immunotoxin is being evaluated in a Phase II clinical trial (NCT00611208), which includes patients with a variety of T cell lymphomas [46]. 

The use of plant-derived toxins in anti-cancer therapeutics is also being explored. Plant toxins can permanently disable protein synthesis in eukaryotic ribosomes by cleaving the 28S rRNA [47,48]. Although plant and bacterial derived toxins have a different mechanism for protein synthesis inhibition, they both can lead to apoptotic cell death. Toxins like ricin, gelonin, saporin-S6, and ebulin 1 have been incorporated in various cancer therapeutics [47,49,50]. One attempt examined whether rituximab’s effectiveness could be enhanced by crosslinking it with saporin-S6. Interestingly, a synergistic effect on protein synthesis inhibition was observed [51]. Additionally, a paper published in 2014 described the identification of pachyerosin as a new ribosomal inactivating protein. This toxin was used to create a novel immunotoxin targeting EpCAM and was demonstrated to be effective in the treatment of liver cancer [52]. The variety of immunotoxins will continue to grow as new ribosome inhibiting proteins are discovered.

## 2. Anti-GPC3 Recombinant Immunotoxins 

Hepatocellular carcinoma (HCC) is the most common form of primary liver cancer [53,54]. It is estimated that worldwide nearly 750,000 people die each year due to liver cancer, with around 50% of these cases occurring in China [55]. Treatment of liver cancer is largely limited to chemotherapy or surgical removal. Chemotherapy is often ineffective because of the liver’s natural resistance to damage by chemicals. Liver cells express ABC transporters capable of exporting a large range of common chemotherapeutic agents which can lead to multidrug resistance [56]. In early diagnosed cancers where surgery is an option, the five-year survival rate ranges from 41% to 74% [57]. If cancer presents in the late stages when surgery is not an option, the only approved chemotherapy treatment is the tyrosine kinase inhibitor, sorafenib [57,58]. This treatment increases the survival rate by only 2–3 months [59,60]. Sorafenib treatment can cause mild side effects that range from fatigue, diarrhea, hand-foot syndrome, hypophosphatemia, and weight loss, to severe side effects that include hypertension, hemorrhaging in the brain, and anemia [59,61,62]. A study on sorafenib determined that the HuH-7 HCC cell line was capable of developing resistance to high dose treatments by modulating the PI3K-Akt signaling pathway [63]. The need for therapeutics that are well tolerated and that exhibit a high level of anti-tumor activity are desperately needed to replace the current options. 

Several targets have emerged for antibody therapies against liver cancer. A search for antibody and hepatocellular carcinoma was conducted on the ClinicalTrials.gov website on 11 April 2016. There were over sixty clinical trials that were identified with this search criteria. These results were narrowed down to the thirty clinical trials that are presented in Table 1 by excluding trials that had not opened yet, excluding trials associated with viral infections or viral testing, and by selecting the most advanced phase for similar drug trials. Antibodies against vascular endothelial growth factor receptor 2 (VEGF-R2) and vascular endothelial growth factor A (VEGF-A) are involved in approximately one third of the current HCC clinical trials. Ramucirumab which blocks the VEGF receptor and bevacizumab which neutralizes the growth factor, are the monoclonal antibodies included in these clinical trials [64,65,66]. Blocking VEGF signaling has been linked to a decrease in vasculogenesis and angiogenesis [67]. This has been attributed to the down regulation of the Ras-Raf and PI3K-Akt pathways [68]. Other targets in HCC include: glypican-3 (GC33), c-Met (onartuzumab), epidermal growth factor receptor (cetuximab), insulin-like growth factor-1 receptor (cixutumumab), insulin-like growth factor I and II (MEDI-573), platelet-derived growth factor receptor A (MEDI-575), activin receptor-like kinase 1 (PF-03446962), endoglin (TRC105), and TROP-2 (sacituzumab) [69,70,71,72,73,74,75]. Additional targets like EpCAM and CD133 are being evaluated in preclinical studies as potential markers for HCC [45,76,77,78]. As HCC is better understood, the list of potential targets will continue to grow. Among these targets, GPC3 is very attractive for therapeutic design because it is uniquely overexpressed in hepatocellular carcinoma [69].

GPC3 represents an exciting opportunity for the development of anti-cancer therapeutics. Research done by Hsu and his colleagues in the late 1990s first showed that GPC3 mRNA was expressed in over 70% of HCC cases [79]. More importantly, GPC3 expression was found to be associated with cancerous cells and not with normal adult liver cells [69,79,80,81]. Research has demonstrated a proliferative effect of GPC3 because it interacts with cell signaling pathways, including the Wnt/Yap pathway [82,83,84]. GPC3 surface expression levels varied between HCC cell lines with Hep3B having one of the highest expressions with an estimated 200,000 sites per cell. HuH-7 and HuH-1 both had considerably less surface expression with around 10,000 sites per cells [85]. Additionally, GPC3 has a high internalization rate which makes it ideal for immunotoxin targeting [85]. The unique expression profile of GPC3 and its association with signaling pathways make it a potential therapeutic target for the treatment of liver cancer.

**Figure 1:**
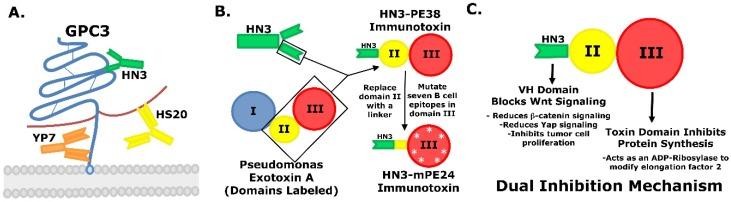
Overview of immunotoxin design. (**A**) Representation of anti-GPC3 antibodies and their approximate binding sites. YP7 and HN3 bind to the core protein near the C-terminus and a conformational epitope that requires both the N-terminus and the C-terminus, respectively. HS20 binds directly to the heparan sulfate chains associated with GPC3; (**B**) Construction of HN3-PE38 and the second generation HN3-mPE24; (**C**) HN3 immunotoxin domains and their associated functions.

Using both hybridoma and phage display antibody technologies, researchers in our lab have identified candidate GPC3 binders. Three antibodies have been selected based on their affinity, epitope location, and anti-tumor activity. A schematic of the antibodies’ approximate binding sites can be found in Figure 1A. The first antibody was isolated using mouse hybridoma technology and was named YP7 [86]. This protein was generated to a C-terminal peptide, so it binds in close proximity to the cell surface. The second antibody generated was a human heavy chain antibody named HN3 [84]. This GPC3 binder was identified from a human single domain antibody phage display library. Unlike a traditional antigen binding region that consists of two domains, this heavy chain antibody only has a single domain in its binding region [84]. The HN3 antibody binds a conformational site on the core protein (Figure 1A) [84]. Its reduced size may prove to be advantageous in tumor penetration. Interestingly, their reduced size allows single domain antibodies to bind epitopes located in protein clefts. These epitopes are generally inaccessible to conventional antibodies due to steric interference [87]. These antibodies may provide access to new binding sites that have not been explored for their therapeutic potential. The third antibody candidate was also isolated using phage display technology. This antibody is a human single chain Fv antibody, which means that the light and heavy chains are translated as a single protein. This antibody was named HS20 and was found to bind directly to the heparan sulfate side chains that post-translationally decorate GPC3 (Figure 1A) [83,88]. These three antibodies demonstrated that they could bind GPC3 with both a level of high specificity and high affinity. 

These three antibodies were used to generate a series of recombinant immunotoxins in order to test their potential clinical applications. Each antibody had their Fv or VH domains fused to domain II and III of the Pseudomonas exotoxin A (PE38). An overview of the HN3 recombinant immunotoxin design can be found in Figure 1B. While the YP7 immunotoxin consistently showed a higher affinity for GPC3 binding, surprisingly it was the HN3 immunotoxin that showed the most effective killing [85]. This observation was not due to the differences in toxin activity because a [^3^H] leucine incorporation assay showed a similar decrease in protein synthesis for both the HN3 and YP7 based immunotoxins [85]. The increased cell cytotoxicity appeared to be related to the inhibition of Wnt/Yap signaling. The proposed dual inhibition mechanism is included in Figure 1C. The fact that YP7 failed to inhibit cell signaling helped to explain why it showed lower cytotoxic activity despite its high binding affinity. The HN3-PE38 immunotoxin showed the greatest ability to inhibit liver cancer growth in mouse xenograft models [85]. A few of the HN3-PE38 treated mice showed complete tumor remission [85]. Interestingly, when HN3-PE38 was given in conjunction with sorafenib, there was no observable increase in cytotoxic activity. However, when HN3-PE38 was paired with irinotecan to inhibit topoisomerase I, there was a significant reduction in tumor size [85]. This research demonstrated that not only could immunotoxins be used as single agents, but also in combination with current chemotherapies. Both the HN3 and HS20 based immunotoxins have clinical potential because of their ability to inhibit cell signaling pathways. However, the ability of HS20 to bind directly to heparan sulfate would suggest that it has the potential to target multiple cancer types that express different glypicans.

## 3. Perspective and Future Directions

The study of PE38 based immunotoxins began to reveal several therapeutic barriers. Issues stemming from off-target toxicity, immunogenicity, and short half-life in the blood stream are all concerns that need to be addressed. Early studies on the optimization of PE38 helped researchers to produce an immunotoxin with significantly reduced side effects. In an attempt to increase proteolytic stability, lysosomal protease cleave sites were identified and removed from domain II. Interestingly, versions that interrupted the eleven amino acid furin cleavage site lost nearly 80% of their cytotoxic ability [89]. When domain II was removed, except for the furin cleavage site, the immunotoxin lost around 50% of its cytotoxicity, but had a ten-fold higher tolerated dose in mice [89]. The removal of domain II resulted in significant reduction of capillary leakage syndrome and decreased pathologies associated with the liver [89,90]. Similar results were observed when the same toxin fragment was incorporated in the HN3-mPE24 immunotoxin [91]. While the immunotoxin’s cytotoxicity was affected, the therapeutic index was greatly increased with the removal of domain II. 

Another therapeutic concern focuses on the foreign nature of the toxin fragment. There is the potential for secondary immune responses generated against the immunotoxin. This can lead to the production of neutralizing antibodies which can reduce the effectiveness of immunotoxin therapy [92]. This generally does not affect the first round of immunotoxin treatment, but can become a significant problem during follow-up treatments. There are currently two proposed strategies for reducing the rate of neutralized antibody formation. One method uses pentostatin and cyclophosphamide to deplete B and T cells during the course of immunotoxin treatment. A study in 2013 used SS1P in combination with pentostatin and cyclophosphamide to treat ten chemotherapy refractory mesothelioma patients. Neutralizing antibody formation was delayed as a result of the pentostatin/cyclophosphamide treatment, with eight of the patients showing no neutralizing antibodies after the first round of treatment [93]. While these results are promising, there is the possibility that anti-tumor T and B cells are also being depleted during treatment. A method that leaves the adaptive immune response intact might be a more favorable option. 

The second method being explored does not deplete the adaptive immune response, but rather reduces the ability of these cells to recognize the toxin fragment as foreign. By removing or mutating the amino acids responsible for eliciting the unwanted secondary immune responses, it may be possible to produce an immunotoxin with greatly reduced immunogenicity. Building upon the truncation of domain II, eight point mutations were made in domain III of the Pseudomonas toxin to silence B cell epitopes. This new design of the SS1P immunotoxin exhibited higher levels of cytotoxic activity against tumor cells in vitro, as well as increased tumor regression in a mouse experiment [90]. Additionally, the sera from five patients that were treated with the original version of SS1P were characterized for their ability to bind the new mutated version. Interestingly, all of the sera showed a decreased ability to bind the mutated version of SS1P, indicating that the B cell epitopes had been effectively silenced [90]. This mutation strategy has helped to increase the effectiveness of immunotoxin therapies in the clinical setting. The success of this strategy has provided evidence that further silencing of epitopes may be required for the production of an optimized immunotoxin. While mutating B cell epitopes will reduce the ability of antibodies to bind, the reduction of T cell activation could significantly reduce the overall immunogenicity. The T20 version being developed has a total of six point mutations to reduce T cell mediated immunogenicity [94]. The strategy for identifying the antigenic fragments responsible for T cell activation is not straight forward. The heterogeneity of the human MHC complexes makes it difficult to determine if a mutation strategy will be 100% effective [95]. We believe that a toxin fragment with mutated T and B cell epitopes may provide the best therapeutic option, but it remains unclear if the immunogenic nature of the immunotoxin conveys any therapeutic benefits. The display of the toxin portion in MHC molecules might promote favorable cytotoxic T cell responses and increased tumor killing. A further study on the tumor microenvironment may be required before the role of immune response in immunotoxin therapy is fully understood. 

There are several other considerations that need to be addressed when point mutations are introduced. One of the biggest concerns deals with the cytotoxic function of the toxin after mutation. If an epitope is part of the catalytic domain or is important for protein folding, then silencing mutations can have a negative effect on immunotoxin function. Additionally, mutations may inadvertently introduce new epitopes that can be targeted by the immune system. This requires new immunotoxins to be screened against existing patient samples to determine the effectiveness of the silencing strategy. It also requires the serum from patients treated with modified immunotoxins to be characterized for anti-immunotoxin antibodies. The ability to produce better therapeutic immunotoxins will ultimately rely on access to patient clinical samples and will constantly be evolving as we learn more about the human immune response. 

The final therapeutic barrier deals with the relatively short half-life of the immunotoxins in circulation. The small size of recombinant immunotoxins makes them susceptible to removal by glomerular filtration in the kidneys [90,96]. The deletion of domain II from the SS1P immunotoxins reduced the already short serum half-life from nineteen minutes to only thirteen minutes [90]. There are several proposed methods for increasing the duration of the immunotoxins in circulations. One method would involve the addition of polyethylene glycol to the recombinant immunotoxins. A study on the anti-microbial protein, Onc112, demonstrated that the half-life of this peptide could be significantly increased after PEGylation [97]. Additionally, this study demonstrated that variation in the size of the PEGylation and the addition of cleavable linkers would contribute to the observed serum half-life [97]. Another approach to help compensate for low serum half-life would be to increase the frequency of immunotoxin injections. The efficacy of this approach was demonstrated in rodent models that used osmotic pumps to continuously inject cytotoxins/immunotoxins to treat arthritis and tumors, respectively [98,99]. Advances in the design, targeting, and administration of recombinant immunotoxins has made this technology an exciting therapeutic option for the treatment of liver cancer and other solid tumors.

## Figures and Tables

**Table 1:** Summary of current clinical trials using antibody-based therapeutics to target hepatocellular carcinoma.

Identifer	Drug(s)	Type	Target	Phase	Status
NCT01911273	PF-03446962	Mono	ALK-1	II	Terminated
NCT01897038	Onartuzumab with Sorafenib	Mono	c-Met/Tyrosine Kinases	I	Completed
NCT01008358	Tremelimumab	Mono	CTLA4	II	Completed
NCT00483405	Cetuximab with chemotherapy	Mono	EGFR	II	Completed
NCT01375569	TRC105	Mono	Endoglin	II	Completed
NCT02560779	TRC105 with Sorafenib	Mono	Endoglin/Tyrosine Kinases	II	Recruiting
NCT01507168	GC33	Mono	GPC3	II	Completed
NCT00639509	Cixutumumab	Mono	IGF-1R	II	Completed
NCT00906373	Cixutumumab with Sorfenib	Mono	IGF-1R/Tyrosine Kinases	II	Completed
NCT02315066	PF-04518600	Mono	OX40(CD134)	I	Recruiting
NCT02595866	Pembrolizumab	Mono	PD-1	I	Recruiting
NCT00966251	Pidilizumab	Mono	PD-1	II	Terminated
NCT02423343	Nivolumab with Galunisertib	Mono	PD-1/TGF-βR1 Kinases	II	Recruiting
NCT01102400	MEDI-575	Mono	PDGFRA	I	Completed
NCT02519348	Durvalumab with Tremelimumab	Mono	PD-L1/CTLA4	II	Recruiting
NCT01308723	RO5323441 with Sorafenib	Mono	PGF/Tyrosine Kinases	I	Completed
NCT01258608	Mapatumumab with Sorafenib	Mono	TRAIL-R1/Tyrosine Kinases	II	Ongoing
NCT00055692	Bevacizumab	Mono	VEGF-A	II	Completed
NCT00467194	Bevacizumab with Rapamycin	Mono	VEGF-A/mTor	I	Completed
NCT01010126	Bevacizumab with Temsirolimus	Mono	VEGF-A/mTor	II	Ongoing
NCT00365391	Bevacizumab with Erlotinib	Mono	VEGF-A/Tyrosine Kinases	II	Completed
NCT00867321	Bevacizumab with Sorafenib	Mono	VEGF-A/Tyrosine Kinases	II	Completed
NCT01140347	Ramucirumab	Mono	VEGF-R2	III	Completed
NCT02069041	Ramucirumab with Oxaliplatin	Mono	VEGF-R2/DNA	I	Recruiting
NCT02572687	Ramucirumab with Durvalumab	Mono	VEGF-R2/PD-L1	I	Recruiting
NCT01498952	MEDI-573 with Sorafenib	Bispecific	IGF-I and IGF-II/Tyrosine Kinases	I	Completed
NCT01631552	Sacituzumab Govitecan	ADC	TROP-2/Topoisomerase	II	Recruiting
NCT00829465	Metuximab labeled with Iodine131	ARC	CD147	IV	Unknown
NCT02723942	T cells expressing αGPC3 Antibody	CAR-T	GPC3	II	Recruiting
NCT02632006	T cells expressing PD-1 Antibody	CAR-T	PD-1	II	Recruiting

Abbreviations: ALK: Activin receptor-like kinase; ADC: Antibody drug conjugate; ARC: Antibody radioisotope conjugate; CAR-T: Chimeric antigen receptor T cell; CTLA: Cytotoxic T-lymphocyte associated protein; EGFR: Epidermal growth factor receptor; GPC3: Glypican-3; IGF(R): Insulin-like growth factor (receptor); Mono: Monoclonal; PD(-L): Programmed cell death protein (-ligand); PDGFRA: Platelet-derived growth factor receptor alpha chain; PGF: Placental growth factor; VEGF(R): Vascular endothelial growth factor (receptor).

## Abbreviations

The following abbreviations are used in this manuscript:

ABC	ATP-Binding Cassette
ADC	Antibody Drug Conjugate
ADCC	Antibody Dependent Cellular Cytotoxicity
ADP	Adenosine Diphosphate
ALK	Activin Receptor-Like Kinase
ARC	Antibody Radioisotope Conjugate
CAR-T	Chimeric Antigen Receptor T Cell
CD	Cluster of Differentiation
CDC	Complement Dependent Cytotoxicity
CTLA	Cytotoxic T-Lymphocyte Associated Protein
EGFR	Epidermal Growth Factor Receptor
EpCAM	Epithelial Cell Adhesion Molecule
ERAD	Endoplasmic Reticulum-Associated Protein Degradation
FDA	Food and Drug Administration
Fv	Variable Fragment
GPC3	Glypican-3
HCC	Hepatocellular Carcinoma
IGF(R)	Insulin-Like Growth Factor (Receptor)
MHC	Major Histocompatibility Complex
MMAE	Monomethyl Auristatin E
Mono	Monoclonal
mPE24	Mutated PE24
mRNA	Messenger Ribonucleic Acid
NCI	National Cancer Institute
PDGFRA	Platelet-Derived Growth Factor Receptor Alpha Chain
PD-1	Programmed Cell Death Protein 1
PD-L1	Programmed Cell Death Protein Ligand 1
PEG	Polyethylene Glycol
PE38	Pseudomonas Exotoxin A Domain II and Domain III
PGF	Placental Growth Factor
RIT	Recombinant Immunotoxin
TCR	T-Cell Receptor
VEGF(R)	Vascular Endothelial Growth Factor (Receptor)
VH	Heavy Chain Variable Region
